# Plant Biology and Biotechnology: Focus on Genomics and Bioinformatics

**DOI:** 10.3390/ijms23126759

**Published:** 2022-06-17

**Authors:** Yuriy L. Orlov, Vladimir A. Ivanisenko, Oxana B. Dobrovolskaya, Ming Chen

**Affiliations:** 1 Agrarian and Technological Institute, Peoples’ Friendship University of Russia, 117198 Moscow, Russia; dobrovolskaya-ob@rudn.ru; 2 The Digital Health Institute, I.M. Sechenov First Moscow State Medical University of the Ministry of Health of the Russian Federation (Sechenov University), 119991 Moscow, Russia; 3 Institute of Cytology and Genetics SB RAS, 630090 Novosibirsk, Russia; salix@bionet.nsc.ru; 4 Department of Bioinformatics, College of Life Sciences, Zhejiang University, Hangzhou 310058, China; mchen@zju.edu.cn

The study of molecular mechanisms of plant stress response is important for agrobiotechnology applications as it was discussed at series of recent bioinformatics conferences [1,2]. Furthermore, it is in the focus of modern sequencing and bioinformatics research [3]. Sequencing-based systems biology approaches offer a comprehensive view of plant growth from molecular to cellular, organ, and population levels [4,5]. Following the demands for the public discussion platform on plant bioinformatics at *MDPI IJMS* we have organized this special journal issue “Plant Biology and Biotechnology: Focus on Genomics and Bioinformatics” (https://www.mdpi.com/journal/ijms/special_issues/Plant_Biotechnology, accessed on 14 June 2022). This issue collected research articles on systems plant biology applications, computational genomics, and bioinformatics methods in plant sciences.

This Special Issue continues the collection of papers published in *MDPI IJMS* “Bioinformatics of Gene Regulations and Structure” (https://www.mdpi.com/journal/ijms/special_issues/Bioinformatics_Genomics, accessed on 14 June 2022) [3] and related journal issues initiated after the series of bioinformatics conferences that were held in Russia—BGRS (https://bgrssb.icgbio.ru/2020/, accessed on 14 June 2022) and Plantgen-21 (14–18 June 2021, Novosibirsk, Russia, https://conf.icgbio.ru/plantgen2021/, accessed on 14 June 2022) [1,2,6]. Some of the studies presented here were reported at the BGRS (Bioinformatics of Genome Regulation and Structure/Systems Biology) traditional biannual computational biology meeting in Novosibirsk, Russia [7,8]. The conference highlights recent advances in genomics, bioinformatics, systems biology, and biotechnology areas [9,10,11]. This collection continues the studies in the field of bioinformatics that were presented initially in the *Frontiers in Genetics* journal [2], in the *PeerJ* journal (https://peerj.com/collections/72-bgrs-sb-2020, accessed on 14 June 2022), and recently at *MDPI Life* special issue [12]. It refers to the topics on computational genomics in model plants for biotechnology, discussing genomics and bioinformatics approaches [13]. High-throughput technologies make it possible to model protein–protein and gene regulatory interactions in plant cells, providing a basis for better crop production and sustainability [14,15,16]. Plant–pathogen interaction studies complement network modeling in this area [5,17].

To note history of the series of post-conference journal Special Issues we refer to Bioinformatics of Genome Regulation and Structure (BGRS) conference series [1,2,6,10,11] and related Schools on Systems Biology and Bioinformatics (SBB) held in Novosibirsk, Russia [7,9,18], later completed by other international conferences on genetics such as Belyaev Conference—2017 [19]. Here we collected works on the topics of gene expression regulation in plants; plant genomics and bioinformatics; plant stress response; miRNA and molecular mechanisms studies in plants; plant systems biology and digital phenotyping.

We open this collection of papers by meta-analysis of molecular mechanisms of plant cell response to sunlight. Aleksandr V. Bobrovskikh and co-authors have presented meta-analysis of light response in *Arabidopsis thaliana* L. [13]. The sensing of high light is complex and includes the plant response at different molecular levels including photoreceptors regulations, chloroplast avoidance movements, accumulation of metabolites, and so on. The authors analyzed genetic systems and candidate transcription factors involved in the response to high light stress in *Arabidopsis thaliana* L. using set of bioinformatics tools. Through the meta-analysis of five transcriptomic experiments the authors constructed a set of differentially expressed genes and transcriptional regulators. This work complements recent systems biology studies of metabolism in Arabidopsis [14,20].

Wheat is import crop to be studied for pathogen resistance by sequencing methods [16,21]. Jana Zwyrtková and colleagues [22] presented analysis of draft sequencing of crested wheatgrass (*Agropyron cristatum*), a wild relative of wheat. The annotation of chromosome-specific sequences characterized the DNA-repeat content and led to the identification of genic sequences. A set of polymorphic simple-sequence-repeat markers was identified. The markers were used to characterize orthologous relationships between *A. cristatum* and common wheat.

Raghav Kataria and Rakesh Kaundal [23] studied the host–pathogen interactome of the wheat–common bunt system. Common bunt, caused by two fungal species, *Tilletia caries* and *Tilletia laevis*, is one of the most potentially destructive diseases of wheat. Using computational approaches millions of probable protein-protein interactions were predicted in *T. aestivum–T. caries* and *T. aestivum–T. laevis* interactomes, respectively. The authors identified 648 and 575 effectors in these interactions, correspondingly. Subcellular localization allowed to suggest that most of the pathogen proteins target the host in the plastid.

Fusarium wilt of flax is an aggressive disease caused by the soil-borne fungal pathogen *Fusarium oxysporum* f. sp. *lini*. It is a challenging pathogen presenting a constant threat to flax production industry worldwide [15]. Alexander Kanapin et al. [24] presented genomics study on Fusarium wilt resistance in flax. The authors performed a genome-wide association study using the collection of the genotypes infected with a highly pathogenic *Fusarium oxysporum* f. sp. *lini* MI39 strain [24]. Several QTNs (Quantitative Trait Nucleotides) were detected; some QTNs spanned regions that harbored genes involved in the pathogen recognition and plant immunity response.

Lidia Samarina et al. [25] studied persimmon (*Diospyros lotus*) in the Northwestern Caucasus based on leaf morphology and multilocus DNA markers. Resistance to cold and frost is important problem for subtropical plants [26,27,28]. *Diospyros lotus* is the one of the most frost-tolerant species in the *Diospyros* genera, used as a rootstock for colder regions. To predict the behavior of *D. lotus* populations in an extreme environment, it is necessary to investigate the intraspecific genetic diversity and phenotypic variability of populations in the colder regions. The results provide a better understanding of adaptive mechanisms in *D. lotus* in extreme environments and will be important for the further expansion of the cultivation area for persimmon in colder regions.

Next papers present methodological works on plant bioinformatics. Eugeniya Bondar and colleagues [29] presented a method for genome-wide prediction of transcription start sites (TSS) in conifers. The aim of this study was to enhance and expand the existing genome annotations using computational approaches for genome-wide prediction of TSSs in the four conifer species: loblolly pine, white spruce, Norway spruce, and Siberian larch. This work provides the basis for future experimental validation and the study of the regulatory regions to understand gene regulation in gymnosperms.

Huan Qi and colleagues [30] studied alternative splicing in plant RNA-Seq data. The authors developed a new bioinformatics tool named ASTool and estimated its functionality in plants. Alternative splicing classification applications have been developed for different genome models [31]. It was noted that intron retention is the most predominant alternative splicing type in plants. ASTool allows detecting and visualizing of novel intron retention events based on known splice sites in plant genes [30].

Inducible transgene expression systems in plants are important for applied research. Evgeniya Omelina et al. [32] reviewed optogenetic and chemical induction systems for regulation of transgene expression in plants. The authors discussed inducible transgene expression systems. It is noted that in contrast with chemical-inducible systems, optogenetic tools enable spatiotemporal, quantitative and reversible control over transgene expression with light, overcoming limitations of chemically-inducible systems.

The molecular mechanisms of plant cell response, as well as gene expression in model plants are studied now based on sequencing technologies and genotyping tools [33]. This Special Issue on plant bioinformatics shows that gene expression regulation studies are on the edge of modern computational biology research [1,2]. The guest editors are happy to announce next BGRS-2022 conference in Russia (https://bgrssb.icgbio.ru/2022/, accessed on 14 June 2022) and the following Special Issue “Bioinformatics of Gene Regulations and Structure—2022” (https://www.mdpi.com/journal/ijms/special_issues/Bioinformatics_Gene, accessed on 14 June 2022) at MDPI IJMS. We wish that the readers find these papers to be interesting and stimulating, and we are continuing to collect papers on gene expression regulation based on novel sequencing and computational approaches, networks models, and pathway analysis [33,34,35].
